# A paradoxical situation in regressivity or progressivity of out of pocket payment for health care: which one is a matter of the health policy maker’s decision to intervention?

**DOI:** 10.1186/s12962-019-0197-0

**Published:** 2019-12-17

**Authors:** Mohsen Bayati, Mohammad Hossein Mehrolhassani, Vahid Yazdi-Feyzabadi

**Affiliations:** 1 Health Human Resources Research Center, School of Management & Information Sciences, Shiraz University of Medical Sciences, Shiraz, Iran; 2 Social Determinants of Health Research Center, Institute for Futures Studies in Health, Kerman University of Medical Sciences, Kerman, Iran; 3 Health Services Management Research Center, Institute for Futures Studies in Health, Kerman University of Medical Sciences, Kerman, Iran

**Keywords:** Financial protection, Kakwani index, Regressivity, Progressivity, Equity

## Abstract

**Background:**

Equity in health financing as one main aspect of health equity plays an essential role on the path toward universal health coverage. Out of pocket payment (OOP), a source with high share to total health expenditure, is an inequitable mechanism for health financing.

**Main body:**

The OOP has been considered regressive (Kakwani index with a negative value) in nature. However, in some studies especially in developing countries, it is reported to be progressive (Kakwani index with a positive value). The main questions are: Is the progressive OOP equitable? What causes this contradiction? What can we do for the proper interpretation? And what are policy implications of this issue? In this commentary we briefly elaborate on these issues. We present several reasons for progressivity of OOP, and several methodological and policy issues for addressing it.

**Conclusions:**

Even if the OOP is progressive and the share of poor people is low, this may financially limit their access to health services, increase their risk of incurring catastrophic health expenditure (CHE), and even pushing them more into poverty. In order to provide a comprehensive picture of equity in health financing, other financial protection indicators such as the redistributive effect, re-rating, exposure to CHE, and impoverishment due to health expenditure should also be estimated and reviewed.

## Background

Financial protection or, in other words, equity in financing is an essential part of universal health coverage. This means that people should not suffer from financial constraints due to payment for health services and protect people who have the least ability to pay [1]. This is one of the main challenges faced by health policy makers in both developed and developing countries [2, 3]. Equity in financing also is one of the key aspects of any health system reforms which is monitored and evaluated during and after each reform [2].

Different indicators are used to assess equity in health financing. One of the main aspects of equity in financing is the vertical focus of financing. To measure vertical equity, researchers use the Kakwani index. This takes a number between − 2 and + 1. The Kakwani index is more positive, the financing is more progressive and the burden of health financing is on the rich [4].

## Paradoxical situation in OOP

Countries use different sources for health financing. One of these is direct out of pocket payment (OOP) when receiving services. Theoretically, the OOP for health care is the most inefficient and inequitable kind of payment, and is quite regressive [5], but sometimes it is a contradiction that given the nature of regressivity of this mechanism, some empirical studies show a progressivity of OOP and this may arise the question of whether it is fair. More precisely, while we know that OOP is always an inherently regressive financing mechanism, what is the fair condition in situation of progressive OOP, which means that the rich are paying a higher OOP than the poor?

Although a positive value of the Kakwani index for OOP indicates that burden of payments at the point of service delivery is concentrated on the rich people, the occurrence of households facing to catastrophic health expenditure (CHE) may ever be remarkable. This issue was indicated in a study reported by World Bank in Mongolia [6].

Now, these questions arise: What causes this contradiction? What are the main consequences? How does the policymaker, on the basis of what criteria, recognize the decision to intervene or take appropriate actions? To answer, first, we provide the various reasons for this contradiction in theory and practice, and then we suggest appropriate policy making in confronting this conflict.

## In which situations will OOP be progressive?

If the poor cannot afford health services at all, or they receive less than their needs, or cheaper services in public sectors (their quality is always one of the challenges, in particular in developing countries), because of their inability to pay, their payments for health services are reduced [7]. So in such a system, relatively poor are paying less than rich people and the Kakwani index has a positive value (progressive). In other words, if the luxury services, more expensive services and more private sector services are used by richer people, payouts should increase from their pockets. So in such a system, the rich are spending more money on their health than the poor.

There is remarkable evidences that indicate progressivity of OOP may be due to this issue that the utilization of health services for rich people has been higher than for poor ones. For example, the results of the study conducted by Chen et al. [8] on financing equity in China’s health care reform showed that the concentration index (CI) and Kakwani index for OOP in both rural and urban areas of Gansu province had a positive value. This indicates a progressivity in financing for OOP and it is concentrated on rich people. However, it can be stated that the CI for the utilization of inpatient and outpatient health services in same areas was also positive which means that the rich people consume the health services more than poor people. In other words, although OOP was progressive, it was observed that the inequality in health service utilization was concentrated on the rich people.

In this situation, people with high socio-economic status (SES) utilize more health services than lower SES (poor people) even in case of services provided by public sector. As shown in Table 1, although the value of CI for different services in public sector such as inpatient, outpatient, nonhospital and even preventive services (HIV test and vaccination) is less than the value of CI for private sector, the values are still positive. This means that inequality is pro-rich and they utilize the health services more than poor people. Implicitly, this shows that many poor people, due to unaffordability, cannot consume their needed services. Thus, because of no consumption or consumption lower than the need, they pay no charge for their health or pay low charge relatively. Other examples are presented in Table 1.

**Table 1:** Evidence on distribution of health expenditure and health service utilization in some selected studies

Country	CI for OOP	Kakwani index for OOP	CI for health care utilization	References
China, Gansu province	Urban: 0.374 Rural: 0.349	Urban: 0.049 Rural: 0.009	Urban (inpatient: 0.328, outpatient: 0.145) Rural (inpatient: 0.331, outpatient: 0.056)	[8]
Mongolia	0.560	0.202	Inpatient admissions (public: 0.013, private: 0.316) Outpatient visits (public: 0.133, private: 0.482) Voluntary counseling and testing for HIV: 0.015 Immunization: 0.013	[6]
Indonesia	0.473	0.176	Hospital-inpatient: 0.424 (public: 0.374, private: 0.495) Hospital-outpatient: 0.341 (public: 0.312, private: 0.381) Non hospital: 0.016 (public: − 0.093, private: 0.090)	[9]
Sri Lanka	0.456	0.068	Hospital-inpatient: 0.011 (public: − 0.055, private: 0.376) Hospital-outpatient: − 0.041 (public: − 0.071, private: 0.126) Non hospital: 0.153 (public: 0.053, private: 0.171)	[9]

*CI* concentration index, *OOP* out-of-pocket payment

We assume that the OOP types including official patient cost sharing, informal payment, and pure private OOP [10], can also affect its progressivity or regressively status. For example, pure private OOP, a payment for which no prepayment (premium/tax) has been made, is mainly done in the private sector and by middle and upper middle socioeconomic classes. On the contrary, OOP under insurance schemes (copayment, coinsurance, indemnity, deductible, cap) is mainly done by people with lower incomes and in public settings. Therefore, OOP in a system with strong insurance schemes or tax system are likely to be consistent with the theory and regressive. Conversely, in systems with high pure private OOP due to the limited benefits package, the fragmented insurance pools, the lack of comprehensive coverage of the entire population, and poor purchasers who deal with few providers on the contract, are likely to change OOP to proportional or progressive status.

## Policy recommendations

In the above cases, although the Kakwani index indicates that the equity in financing is progressive and suitable, in reality there is injustice. Therefore, for a more realistic and deeper understanding of the issue, we propose:

- In addition to the ongoing assessment of equity in health financing, equity in utilization of health services be regularly monitored and audited. In this regard, the results of these evaluations determine whether the progressivity status of the OOP is relevant to less/more service utilization by the poor/the rich people [11]. Please refer to some researches depicted in Table 1.
- The Kakwani index to be estimated for various health services. In this case, it specifies which services are responsible for this issue (progressivity of OOP): general practitioner visits, specialist visits, medications, hospital services, dentistry services and so on? We can state that the demand for services with different income and price elasticity can affect the behavior of people in receiving services based on people’s income, the cost of that service, and the amount of OOP [12].
- Continuous inclusion and updating the benefits package is an interactive process between the laws governing the health system for providing essential services and the use of robust evidence to monitor changes and preferences of the community [13]. So it is suggested that the Kakwani index be calculated for the basic and priority health services, which is the responsibility of the government (and equity in their financing is pivotal). Moreover, the index is separately measured for payments concerning luxury medical and dentistry services, brand medications and private hospitals. Their comparison will be the basis for deciding on coverage of services by supplementary health insurance. For example, if cosmetic surgeries irrespective of those surgeries that are aimed at reducing social stigma and complications such as burns, they are considered to be a luxury service, increase the OOP in rich people. This issue, with the exception of matters related to social health, does not undermine the concern of equity. Therefore, the data must be collected in such a way as to enable the analysis of the causes and the partial interpretation of factors affecting the OOP.
- Calculating the Kakwani index for OOP by its different types signifies its role on progressivity or regressively of the index. Therefore, more precise, relevant and targeted policies can be formulated and implemented. Finally, in the use of the Kakwani index for equity in the health financing, especially in OOP, there are several considerations to be taken into account. In addition, in order to provide a comprehensive picture of equity in health financing, other indicators such as the redistributive effect, re-rating, exposure to CHE, and impoverishment due to health expenditure should also be estimated and reviewed.

## Conclusions

In summary, OOP, which are always an inequitable financing mechanism, can challenge both the rich and poor in paying for health services. Even if this financing mechanism does not directly impede receiving the needed health services, it may potentially lead to some adverse effects such as selling durable assets and borrowing the money to buy health services that aggravate the poverty, exacerbate financial hardships or shift the cost behavior of households (limiting cost in other household expenditures baskets such as housing, savings, communication and transportation and recreations). This may have some adverse effects on the health of households and strengthens the cycle of disease and poverty [14, 15]. By the same token, although OOP is progressive and the sharing of financing for the poor is low, this low share may limit their access, incur catastrophic expenditure or even push them more into poverty [7].

The policy maker’s decision to take appropriate interventions to reduce inequity in health financing is dependent on a continuous, scrupulous, detailed and regular evaluation of the all indicators of financial protection and equity in service utilization together. Of course, it is important to note that merely measuring inequity in the area of health and its subsequent equity-based decisions do not result in the improvement of equity-centered outcome in the health system. In some cases, despite the regressive tax based system, there are some difficulties to meet the requirements to institutionalize a new reform for moving forward to less regressive or more progressive financing system. Most important of these difficulties are including the limitation to finance costs or other required resources for implementation of new mechanisms, considerations related to degree of socioeconomic development (the limited income capacity of the country and households), low fiscal capacity to mobilize enough money to meet the desired level of expenditures in the health sector (many workers in the informal sector of economy), and lack of enough political accountability (the degree of democratic political system and the authority over the financing process that allows citizens appropriate control over the process.). Thus, it is not surprising if in some cases a less progressive financing system collects and pools resources better than a more progressive tax-based system or a progressive tax-based system may face with difficulties in providing the costs of health system due to unwillingness to pay tax by rich people. In conclusion, equity in financing is a contextual process dependent on the analysis of conditions regarding a wide range of social, technological, economic, environmental, political, and cultural factors of countries.

## Data Availability

Not applicable.

## Abbreviations

CHE: catastrophic health expenditure
CI: concentration index
OOP: out of pocket payments
SES: socio-economic status
